# Genomics, population dynamics, immune evasion and resistance determinants foster the competence and global dissemination of *Klebsiella pneumoniae*

**DOI:** 10.7717/peerj.20296

**Published:** 2025-11-28

**Authors:** Bilal Aslam, Sulaiman F. Aljasir

**Affiliations:** Department of Veterinary Preventive Medicine, College of Veterinary Medicine Qassim University Buraydah, Buraydah, Saudi Arabia

**Keywords:** *K. pneumoniae*, Multi-drug resistant, Immune evasion, Resistance determinants, Population dynamics, Global dissemination, Public health, Resistant infection

## Abstract

**Background:**

According to the World Health Organization (WHO), *Klebsiella pneumoniae* is a critical public health concern and an established ESKAPE (*E. faecium*, *S. aureus*, *K. pneumoniae*, *A. baumannii, P. aeruginosa*, and *Enterobacter* spp.) pathogen. Mounting incidence of MDR *K. pneumoniae* is worrisome across the globe. *K. pneumoniae* is an established ubiquitous pathogen and associated with various infections in a wide range of hosts.

**Methods:**

The peer reviewed findings with given problem statements were thoroughly studied through literature review technique. Multiple antibiotic-resistance genes and virulence genes across various Klebsiella species were studied to explore their evolutionary dynamics and genetic diversity.

**Results:**

Population dynamics revealed that the clonal group (CG) 258 and CG 14 are considered as global disseminated clones. The genome size (5.7 Mbps) of *K. pneumoniae* is reported to be larger than the other Enterobacteriaceae which allows *K. pneumoniae* to survive in diverse geographical niches. It has adequate resistome and virulence machinery to evade the host immune system and establish the infection. Due to the emergence of resistant variants *K. pneumoniae* needs appropriate alternative control measures.

**Conclusion:**

The current review described the characteristics features of *K.* *pneumoniae* which are the key players in making this organism a credential pathogen. Additionally, it would be instructive and underpin the molecular insights that may aid in restraining this pathogen.

## Introduction

The efficacy of antibiotics in modern medicine is increasingly undermined by the rise of antimicrobial resistance (AMR), with *Klebsiella pneumoniae* recognized as a member of the ESKAPE group responsible for the majority of antibiotic-resistant hospital-acquired and community-acquired infections. This pathogen is associated with severe, often life-threatening infections, and treatment options remain limited. According to the World Health Organization (WHO), *K. pneumoniae* is listed among the highest priority critical public health threats (Wyres & Holt, 2018). Its high genomic diversity and prevalence of antibiotic resistance genes (ARGs) further amplify its clinical importance.

From a resistance perspective, *K. pneumoniae* not only poses a direct therapeutic challenge but also serves as a significant reservoir and disseminator of ARGs, including *bla*KPC, *bla*NDM-1, and *bla*OXA-48. The spread of these genes occurs *via* vertical transmission or horizontal gene transfer mediated by mobile genetic elements (MGEs) such as plasmids, integrons, insertion sequences (IS), and transposons (Navon-Venezia, Kondratyeva & Carattoli, 2017). These combined features of *K. pneumoniae*, clinical severity, resistance gene diversity, and efficient dissemination underscore the urgent need for intensified research and surveillance (Aslam et al., 2022; Wyres & Holt, 2018; Al-Ouqaili, 2018a).

During the last decade, *K. pneumoniae* has emerged as a substantial health concern due to the increasing incidence of MDR *K. pneumoniae* infections across the globe. Some *K. pneumoniae* strains known as hypervirulent (hypermucovisous) variants present an additional agitating mechanism of hyper-virulence due to the acquired virulence factors, first reported in Asia in the 1990s and now have been reported all over the world. *K. pneumoniae* plays a central role in the global antimicrobial resistance crisis; the existing data advocates that it has a greater ecological range, significantly diverse composition of DNA, ARG diversity, and plasmid liability than the other Gram-negative bacilli (GNB) (Aslam et al., 2021b; Wyres, Lam & Holt, 2020).

*K. pneumoniae* infections need controlling measures such as prompt diagnosis, detection and containment of resistant variants, improved vaccine production, and use of alternative treatment approaches like phage or immunotherapy (Aslam et al., 2021a, 2018; Wyres, Lam & Holt, 2020; Xiao, Wu & Dall’Acqua, 2016; Hussein, Al-Kubaisy & Al-Ouqaili, 2024). However, all the above-mentioned containment measures still failed due to the diverse nature of *K. pneumoniae*.

Therefore, there is an urgent need for appropriate novel therapeutic and controlling measures. In this script, we present the taxonomic and genomic characteristic features of *K. pneumoniae*, which are the key players in making *K. pneumoniae* a credential pathogen. Further, we highlight the transmission mechanism, infection biology, and immune evasion of *K. pneumoniae*.

## Rationale

Antimicrobial resistance (AMR) is a pressing global health threat, with multidrug-resistant pathogens undermining modern medicine and straining healthcare systems worldwide. Understanding the mechanisms that render bacteria resistant to antibiotics is crucial and may play a key role in addressing this challenge. As a member of the ESKAPE group, *Klebsiella pneumoniae* poses a substantial health and economic burden globally. It exhibits complex molecular mechanisms associated with resistance, virulence, and immune evasion. A clear understanding of these factors is critical for identifying viable solutions to this global concern. Accordingly, this manuscript is intended for scientists, clinicians, and researchers in molecular biology and microbiology.

## Search methodology

Peer-reviewed studies addressing the identified problem statements were thoroughly examined through a comprehensive literature review. This approach enabled the identification of explicit insights, research gaps, and future perspectives related to the pathogen, which are presented in this manuscript. Electronic databases, including Web of Science, ScienceDirect, Scopus, PubMed, and Google Scholar, were extensively searched using multiple keywords such as *Klebsiella pneumoniae*, population genomics, and multidrug-resistant *K. pneumoniae*. These databases were selected for their scientific credibility and broad subject coverage. This rigorous search strategy ensured the relevance, clarity, and precision of the information compiled in this review.

## Taxonomy

The genus Klebsiella is designated after the name of a German microbiologist named Edwin Klebs in 1885, who later described the species *Klebsiella pneumoniae* in 1887 (Martínez et al., 2004). The causative agent of opportunistic infections belongs to the family Enterobacteriaceae (Partridge et al., 2018). Historically, Friedlander identified a pathogen from the patient’s lungs that died due to pneumonia (Ashurst & Dawson, 2023; Friedländer, 1882). Later in that decade two scientists came up with descriptions for the Friedlander bacterium and named it *Hyalococcus pneumoniae* (Ashurst & Dawson, 2023). Klebsiella was first described by a patient suffering from rhino scleroma later this organism was named “Klebsiella rhinoscleromatis”. In the post-antibiotic era, the most prominent and widely cited efforts were made by different scientists such as Cowan in 1960, Bascomb in 1971, Buchanan and Gibbons in 1974, Brenner in 1977, Woodward in 1979, Izard in 1981, Bagley in 1981 and Naemura in 1979 discovering and arguing the taxonomic position of previously discovered species, concluded different groups within the genus as: (i*) K. pneumoniae* including *K. ozaena* and *K. rhinoscleromatis* from clinical origin, (ii) *K. oxytoca* from environmental and clinical origin, (iii) *K. terrigena* and (iv) *K. planticola* from soil and botanical origin, respectively (Trevisan, 1887).

The phylogenetic analysis based on the 16SRNA subunit conducted in 2003, the Generic division of Klebsiella contains closely linked clusters. Klebsiella are much more related to each other than the neighboring bacterial clusters such as Serratia and Citrobacter (Boye & Hansen, 2003). Based on the whole genome and gyrA sequences of *K. pneumoniae* clinical isolates, it split into three distinct species, *K. pneumoniae* (KpI), *K. quasipneumoniae* (KpII), and *K. variicola* (KpIII). Further, it has been demonstrated that *K. pneumoniae* (KpI) is mostly related to human infection (Holt et al., 2015). Substantial genetic divergence among the species, as indicated by the numerical values on the branches such as 8.89, 0.46, and 9.03, which measure the genetic distances or evolutionary changes. Species like *K. pneumoniae* and *K. varricola* are shown to cluster closely (*K. pneumoniae* complex), suggesting a more recent common ancestry compared to other species such as *K. oxytoca* and *K. michiganensis*.

**Figure 1:**
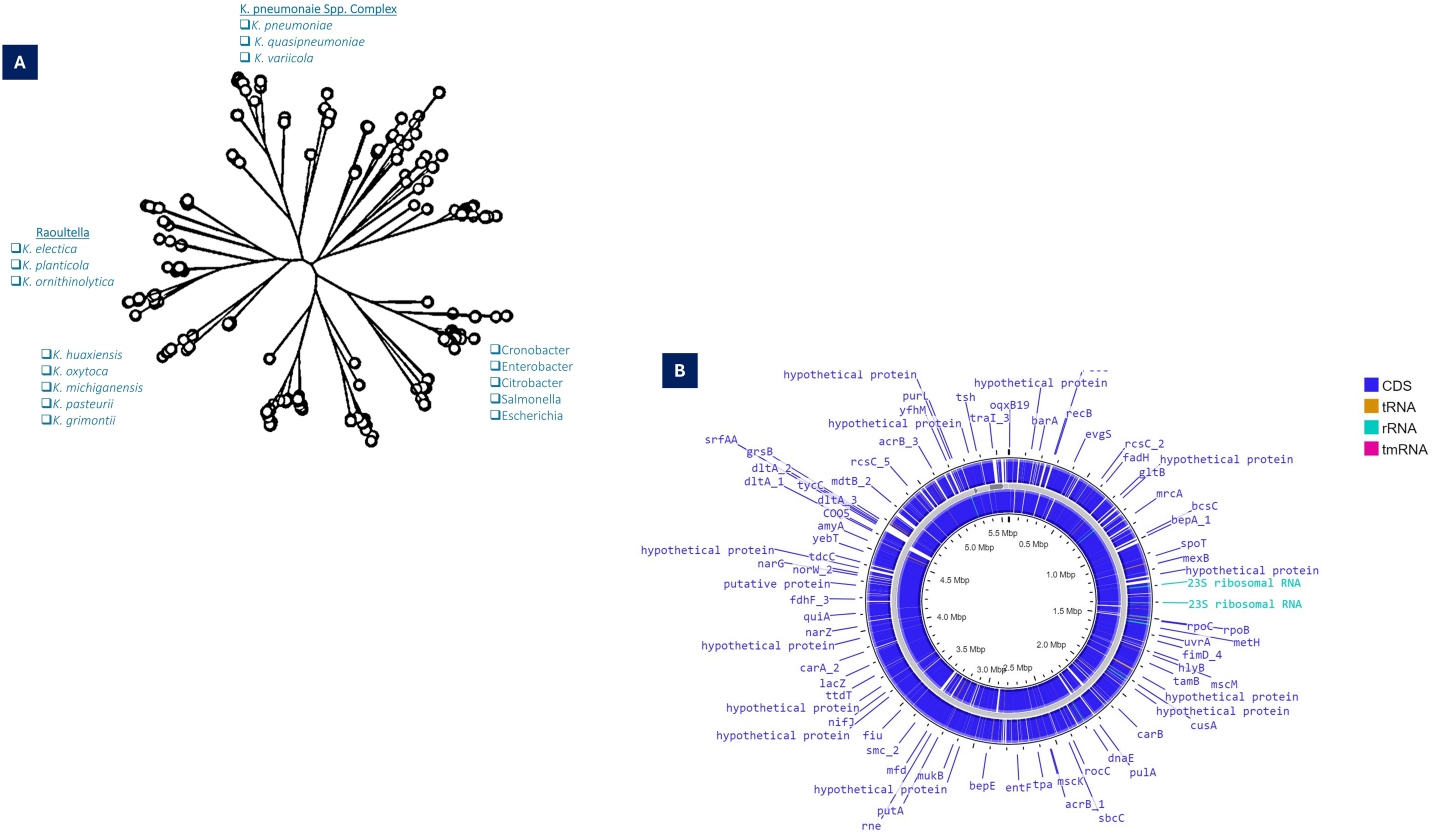
(A) Taxonomy details (Phyloviz) of *K. pneumoniae*, along with the positioning of different *Klebsiella* spp. (B) Circular genomic orchestrate of *K. pneumoniae*, showing genetic, virulence and resistance determinants.

For *K. pneumoniae*, this clustering indicates not only the evolutionary pathways of these bacteria but also their adaptation strategies to different environments or hosts (Fig. 1A).

The WGS revealed that KpI and KpII are equally virulent as both species have acquired the *K. pneumoniae* carbapenemase (KPC) gene and the New Delhi metal-lo-beta-lactamase-1 (NDM-1) gene (Long et al., 2017) (Fig. 1B). With genome-wide average nucleotide identity (≥3%) these closely related phylogenetic species are collectively designated as *K. pneumoniae* species complex (KpsC) (Bialek-Davenet et al., 2014a). Other KpsC included *K. quasipneumoniae subsp. similipneumoniae* (Kp4), *K. variicola subsp. Tropica* (Kp5) (Barbier et al., 2020), *K. quasivariicola* (Kp6), *K. africana* (Kp7) (Long et al., 2017). Like *K. pneumoniae, K. variicola* and *K. quasipneumoniae* are also commonly found bacteria in nosocomial infections (Potter et al., 2018). These KpsC are emerging threats to hospitalized patients as they can acquire resistance plasmids from *K. pneumoniae* (Mathers et al., 2019; Rodríguez-Medina et al., 2019). The *Klebsiella pneumoniae* species complex (Kp) comprises a group of closely related bacteria that were historically classified as a single species (*K. pneumoniae*). Advances in molecular taxonomy have revealed that this complex consists of seven distinct phylogroups (Kp1–Kp7), differentiated based on genomic analyses. Kp1 corresponds to *K. pneumoniae sensu* stricto (the “true” *K. pneumoniae*), whereas Kp2–Kp7 represent separate but closely related species, including *K. quasipneumoniae*, *K. variicola*, and others (Barbier et al., 2020). Phenomena for evolution “descent with modification” allows microbes of a population to adapt and survive within the vast range of habitat in exposure to selective or environmental pressure and severe use of antibiotics-induced selective pressure, which resulted in the geographical distribution of mutated clones (Al-Ouqaili, 2018b; Pitout & Finn, 2020).

## Population dynamics

**Figure 2:**
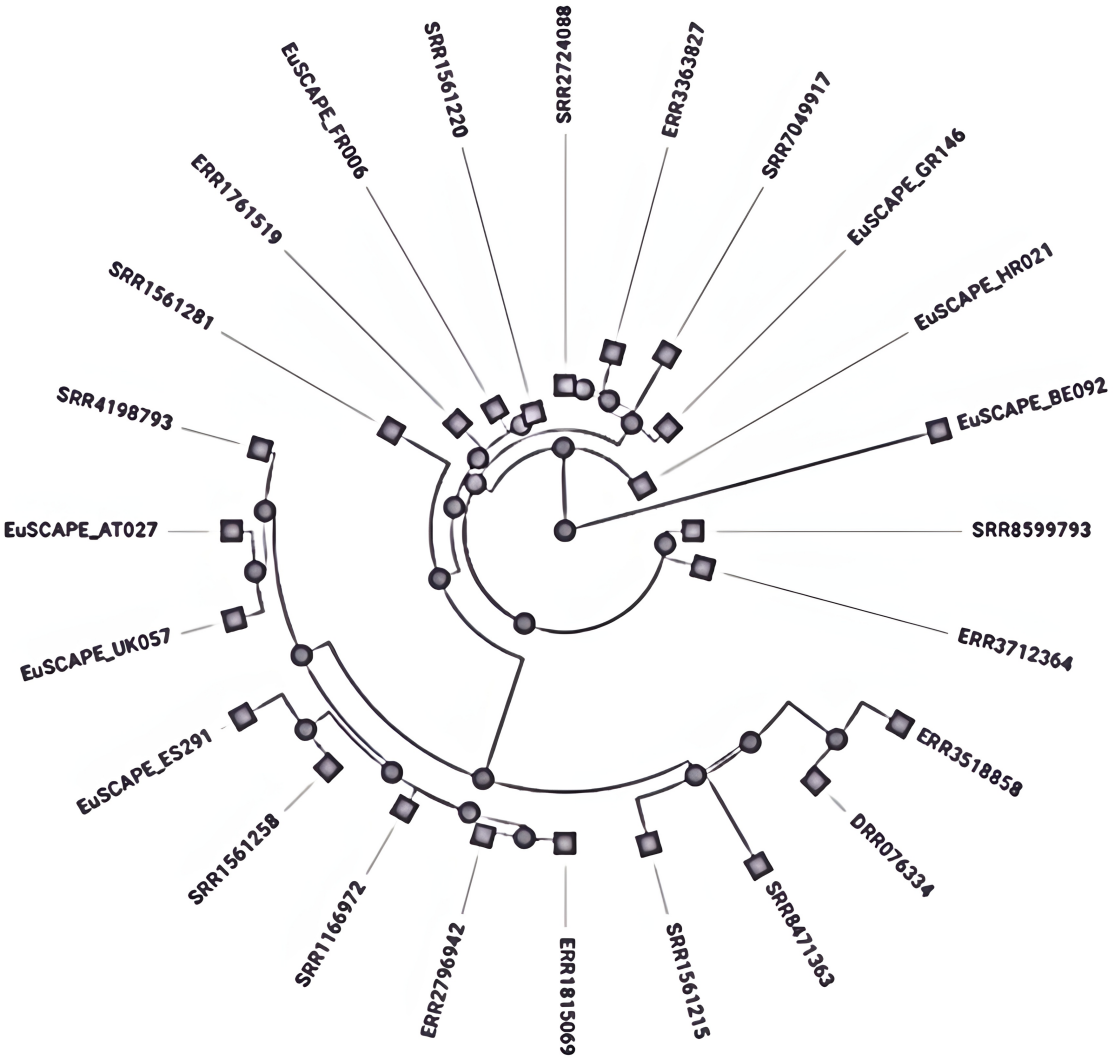
Phylogenetic tree showing the relative depth of the (CG258) nodes extracted from Kleborate, Pathogenwatch.

Different mechanisms have been reported for subtyping the *K. pneumoniae*, MLST is the most widely used method which employs sequencing of seven core genes named rpoB, gapA, mdh, pgi, phoE, infB, and tonB to check variation within these genes and given numerical numbers to each different sequence alleles set the sequence type (ST) (Brisse et al., 2009). The closely related sequence types whose gene sequence differences occurred by point mutation and have a similarity of 90–98% are combined to form a clonal complex (CC) by using the eBURST program (Turner et al., 2007). Further, these CCs have been arranged into subsets called clonal groups (CGs) containing central genotypes along their single-locus variants (SLVs). The CGs are termed according to the central ST, which was selected for the definition like CG258 is named due to its central genotype *i.e*., ST258 (Breurec et al., 2013). These clones are the main source of antibiotic resistance and are referred to as High-risk (HiR) clonal groups with the ability to transfer the resistance genes (Baker & Thomson, 2018). *K. pneumoniae* clonal group CG 258 (ST258, ST11, 83 ST512) and CG14 (ST14 and ST15) are considered global disseminated health threats (Breurec et al., 2013) (Fig. 2). Recent reports have indicated that *K. pneumoniae* ST307 and ST147 are emerging global clones (Peirano et al., 2020), first reported in the USA with blaKPC-2 (Castanheira et al., 2013) and in Pakistan blaCTX-M-15 (Castanheira et al., 2013) and later appeared with blaOXA-48 (Ruiz-Garbajosa et al., 2016). After 2016 the recombination of hypervirulent (HvKP), carbapenem-resistant *K. pneumoniae* isolates produced a superbug of epidemic potential (Chen et al., 2004). Among these CG 23 contains K1-type hypervirulent isolates, whereas K2 type is scattered among various clonal groups immensely (Baker & Thomson, 2018). However, both K1 and K2 types are the most common HvKP with epi-demic potential (Brisse et al., 2009).

Other hypervirulent *K. pneumoniae* K-types included K5, K20, K54, and K57 (Zhang et al., 2016). All isolates within GC 23 are hypervirulent among these ST23, ST26, ST57, and ST163 are of epidemic potential (Zhang et al., 2016), whereas the hypervirulence associated genes were generally encoded by MGEs, including the integrative conjugative element (ICE) (Lam et al., 2018a). Two large resistance plasmids pLVPK from CG43 (Peirano & Chen, 2020) and pK2044 from K1 types (Wu et al., 2009) contain hypervirulence signature genes, including rmpA and/or rmpA2 (regulators of the mucoid Phenotype), iro (salmochelin) and iuc (Aerobactin) siderophores (Wu et al., 2009).

Several plasmids are prevalent in different clonal groups like CG23, CG86, CG65, CG66, and CG380 (Lam et al., 2018b). *K. pneumoniae* carbapenemases genes like blaKPC, blaNDM, and blaOXA and their dissemination within STs and various GCs is a substantial concern. Populations of CG 258 are considered a main vehicle for the pandemic expansion of blaKPC-harboring *K. pneumoniae* (Munoz-Price et al., 2013) and blaNDM is frequently associated with ST11, ST14, ST147, ST149 and ST231 (Tängdén & Giske, 2015). While global dissemination of blaOXA-48-harboring *K. pneumoniae* is associated with mobile element Tn1999 (Poirel, Potron & Nordmann, 2012) and frequently prevalent in several STs *e.g*., ST11 and ST405, *etc*. (Fang et al., 2007). Isolates belonging to GC258 and ST258 & ST512 are the common cause of HAIs (Poirel, Potron & Nordmann, 2012), whereas isolates from GC 23, CG65, and CG86 are associated with invasive community-acquired infections (CAIs) (Decré et al., 2011; Munoz-Price et al., 2013). A detailed description of various CGs along with their STs and virulence determinants *etc*. is given in Table 1.

**Table 1:** Clonal dissemination. Regional distribution of *K. pneumoniae* clonal groups.

Endemic countries	CGs	STs	Dominant K & O locus	GC content %	Virulence determinants	Resistance determinants	MGEs	Type of infection	References
Singapore, Vietnam, Russia,	CG23	ST23, ST26, ST57 and ST163	KL1, O1v2	56.6–57.2	ybt 1, clb 2, iuc 1, iro 1, (RmpADC/rmpA2), rmp 1; KpVP-1/rmpA2, iucABCD-iutA	CTX-M-15 ESBL and *bla*_OXA-48_, Mutations in gyrA or parC, sul1 tetAr	IncA/C_2_, IncFIB (pQil), IncFIB, IncX_3_, ColRNAI, and Col440II	Pneumonia, bacteremia, sepsis, abdominal infection, liver abscess and invasive infections	Brisse et al. (2009), Livermore et al. (2020), Shankar et al. (2020), Lam et al. (2018b)
Madagascar, China.	CG380	ST375	KL2, O1v2	57.1–57.5	ybt 1, ybt 14, iuc 1 iro 1, (RmpADC/rmpA2,	blaKPC-2 blaSHV-11, SHV-1	IncL/M plasmid	Meningitis, liver abscess, severe CAI, invasive infection in diabetic patients	Bialek-Davenet et al. (2014b), Zhan et al. (2017), Magiorakos et al. (2012)
Singapore, Vietnam	CG65	ST65	KL2, O1v2	56.8–57.2	(RmpADC/rmpA2), ybt 17, clb 3, iuc 1, iro, iucABCD-iutA, entB, wabG, uge and ycfM,	blaKPC-2 blaSHV-11, SHV-1, blaKPC-3, *SHV-1*		UTI’s pneumonia, septicemia, liver abscess, invasive infections, CAI’s	Magiorakos et al. (2012), Zhan et al. (2017)
Vietnam, New Zealand, Australia	CG86	ST86	KL2, O1v1	56.5–57.5	ybtS, iucABCD-iutA, rmpA and entB	SHV-1	IncL/M plasmid	Invasive infection, sepsis, liver abscess, CAI’s	Zhang et al. (2016), Surgers et al. (2016), Magiorakos et al. (2012)
United Kingdoms, United States of America, Vietnam	CG25	ST25, ST277,ST326,ST309	KL2, O1v2	57.1–57.4	ybt 2, ybt 16, ybt 9, ybt 6, 3, iro 3, iucABCD-iutA	SHV-1 CTX-M 15 OXA-48	IncFII IncFIB ColKP3	UTI’s septicemia, pneumonia, liver abscess	Breurec et al. (2013), Potron et al. (2013), Navon-Venezia, Kondratyeva & Carattoli (2017)
United Kingdoms, United States, Netherlands	CG37	ST37	KL15, KL12, KL38. O2v2 O3b, O4, OL103	56.7–57.4	ybt 3, ybt 5, ybt 9, ybt 14 (RmpADC/rmpA2),	OXA-48 TEM-1, SHV-11 OXA-48, KPC-2, KPC-3, OXA, NDM, CTX-M15	pKPN-704 pKPN-332	UTI’s, RI’s, septicemia,	Zaman et al. (2018), Wijetunge et al. (2014), Navon-Venezia, Kondratyeva & Carattoli (2017), Liu et al. (2017)
United Kingdoms, Serbia, Romania Netherlands, Italy	CG101	ST101	KL17, O1v1	56.3–56.9	ybt 9, (RmpADC/rmpA2), clb 3, iro1	blaKPC−2, KPC-2, KPC-3, OXA-48, NDM, CTX-M-15, OmpK35/OmpK36	Tn1721 transposon, IncFII(K), IncR, IncFIB, IncFII, IncQ1, and Col440II	Blood stream infections, HAI’s, UTI’s,	Breurec et al. (2013), Loconsole et al. (2020), Roe et al. (2019)
United Kingdoms, United States, Thailand, Russia, Oman, Netherlands, Pakistan	CG147	ST147,ST392	KL19, KL64, O2v1, O3/O3a	56.4–57.4	ybt 9, ybt 16, (RmpADC/rmpA2),	NDM-1, NDM-9, ARMA, AADA1, AAC(6′)-IB, APH(3′)-VI, APH(3′)-1A, CATB3, DFRA5, MPH(E), MSR(E), QNRS1, SUL1, SUL2, CTX-M-15, OXA-1, OXA-9, TEM-1A	IncF, IncA/C and IncL/M, pKpQIL, pKPN3, pNDM-MAR and IncR IncA/C, ColRNAI	Nosocomial infections, abdominal wound infections, UTI’s	Falcone et al. (2020), Lee et al. (2016), Samuelsen et al. (2011), Ouertani et al. (2016)
Pakistan, United States, United Kingdoms, Vietnam, Spain, Netherlands, Nepal, Germany, China.	CG15	ST15	KL24, KL112. O1v1	56.6–57.4	ybt 1, ybt 16, ybt 13 iuc 3, clb 3	KPC-2, KPC-3, OXA-48, NDM,CTX-M, aac(3)-IIa, aph(3′)-Ia, blaOXA-48, MgrB, tet(A), catA1,	IncQ, ColRNAI, IncL, ColpVC, and IncFIB, IncFII	Pediatric infections, UTI’s, neonatal meningitis	Lee et al. (2016), Martins et al. (2020), Pillonel et al. (2018), Löhr et al. (2015)
United States, Italy, Greece, Germany, Australia, Israel China, Spain, United States, Brazil	CG258	ST11,340, 258, 512	ST258 [KL106, KL107, O2v2] ST11 [KL105, KL24, KL15, KL47, KL64. O2v1, O2v2, O3b, O4,OL101]	56.7–57.4 56.9–57.4	ybt 14, ybt 13 ybt 17, clb 3, iucABCD-iutA	blaKPC-2 blaSHV-11, blaKPC-3, bla OXA-9, CTX-M-15, SHV-1, SHV-11, SHV-12), blaOXA-48 frame shift mutation in mgrB, mcr, aph3-Ia	ICEKp258.1 and ICEKp258.Tn4401	Neurosurgical site infections, urinary tract, bacteremia, lower respiratory tract infections, surgical intensive care unit infections, pneumonia	Chen et al. (2014), Kitchel et al. (2009), Fasciana et al. (2019), Wyres, Lam & Holt (2020), Ojdana et al. (2020)
United States, United Kingdoms, Norway, Netherlands, Italy	CG307	ST307	KL102, O2v2	56.6–57.3	(RmpADC/rmpA2), (T4SS), mobA and mobB, ybt, irp1, irp2 and fyuA, π-fimbrial chaperone/usher pathway.	*acc3, blaSHV, blaCTX-15, bla*_KPC-3_, *bla*_NDM-1_, *bla*_OXA-48_, and *bla*_CTX-M-15_, KPC-3, KPC-2, aac(3)-IIa, aac(6′)Ib-cr, qnrB, tet(A), strAB, sul2, dfrA14 and catB3, SHV-28, oqxAB and fosA	pKPN-307 Tn1721 FIB-M, HIB-M, FIBK, FIIK, pKpQIL, IncN type B, n5403-ΔISKpn6-bla KPC-2–ISKpn7	Sepsis, UTI’s, Pneumonia, Neonatal Infections	Villa et al. (2017, 2016), Haller et al. (2019)
Thailand, United States, Netherland, Australia,	CC16	ST16	KL51, O3b	56.9–57.5	ybt 9, ybt 1, (RmpADC/rmpA2),	qnrS, rmtB, mphA and bla OXA-181, bla OXA-48, arr3, catA, aadA16, rmtB, sulI, mphA, bla TEM-1, bla CTX-M-15, dfrA, qnrS, qnrB, tetA, mutations on gyrA and parC, Disruption mgrB gene by an ISL3-like insertion sequence	IncFII, ISL3-like insertion sequence, IncL plasmid, ISL3-like element, Col(pHAD28)/Col440II, Col(IRGK),	Super infections, VAP, blood stream infections, meningitis, septic shock, sepsis, pneumonia	Nguyen et al. (2021), Boonyasiri et al. (2021), Nguyen et al. (2021)
Croatia, Spain	CC 11	ST 437	KL36, O4	57.2–57.5	Ybt 1, rmpA (RmpADC/rmpA2)	KPC-2, blaOXA-232, CTX-M-15, blaNDM, blaCTX-M-55, aph (3′)-IIa, aph (3″)-Ib, aph (6)-Id, and rmtB, oqxA and oqxB, sul2, (floR), (tetA), OXA-9, TEM-1	Tn4401b, IncN, ISKpn7, ColKP3-type no conjugative plasmid, IncFIB (K), IncR, Col440I, IncFII (K), IncP1.	Community acquired urinary tract infections, nosocomial infections.	Francisco et al. (2019), Weng et al. (2020), Fuster et al. (2020)
China	CC1571	ST4564			iucA, iutA, rmpA, rmpA2 and iroN, magA, iutA, fepD, iroE, acrAB, rcsAB, T6SS	blaCTX-M-14, blaCTX-M-17, acrA, acrB, NDM-1 and CTX-M-9, mcr-1, blaNDM, blaTEM, qnrBs, mphA, mrx, sul1, sul2		HAI’s	Wang et al. (2021)

## Genome composition

Comparative genomic studies of *Klebsiella pneumoniae* isolates reveal substantial inter-strain diversity, with phylogenetic analyses identifying ~0.5% sequence divergence between major lineages (Bialek-Davenet et al., 2014a). The species’ remarkable adaptability stems from a dual genomic architecture: a set of approximately 2,000 universally conserved genes maintaining essential cellular functions, coexisting with an expansive variable genome. Individual strains typically carry ~3,500 accessory elements drawn from a shared pool exceeding 30,000 non-core genes (Holt et al., 2015). This genetic repertoire enables the production of over 100,000 distinct protein-coding sequences across the species, facilitating rapid environmental adaptation and clinical persistence (Vernikos et al., 2015; Wyres, Lam & Holt, 2020).

Functional annotation of *K. pneumoniae* genomes reveals distinct enrichment patterns, with membrane transport systems representing 13% of coding sequences, carbohydrate metabolism accounting for 19%, and other metabolic functions comprising 18% of the genetic repertoire (Blin et al., 2017). From a genomic architecture perspective, *K. pneumoniae* possesses a median genome size of 5.7 megabase pairs containing 5,455 predicted open reading frames—significantly larger than related Enterobacteriaceae. For comparison, *Escherichia coli* strains average 5.1 Mbp with 4,915 genes, while *Enterobacter cloacae* isolates typically maintain 5.0 Mbp genomes encoding 4,680 genes. This expanded coding capacity correlates with enhanced environmental adaptability observed in clinical and natural settings (Fig. 1B). Genomic GC content serves as a fundamental taxonomic marker, with significant variation observed between bacterial species (Mann & Chen, 2010). In *Klebsiella pneumoniae*, comparative analyses demonstrate striking differences between core and accessory genome components—while conserved core genes maintain a stable 58% GC ratio, horizontally acquired elements exhibit substantial variability (20% to >70%), reflecting their diverse phylogenetic origins (Holt et al., 2015; Wyres, Lam & Holt, 2020). This genomic heterogeneity exceeds that observed in *Escherichia coli*, supporting the exceptional propensity of *K. pneumoniae* for horizontal gene transfer (McInerney, McNally & O’Connell, 2017). Phylogenetic reconstruction of accessory genes implicates acquisition events from numerous bacterial families, with donor lineages spanning *Enterobacteriaceae* relatives to evolutionarily distant genera such as *Burkholderia* and *Streptomyces* (Holt et al., 2015). Molecular epidemiology studies further confirm shared plasmid pools between *K. pneumoniae* and related enteric species, including *Citrobacter* and *Enterobacter*, demonstrating active interspecies genetic exchange networks (Conlan et al., 2016; Martin et al., 2017; Sheppard et al., 2016).

## Virulence factors

Hypervirulent (hvKp) strains of *Klebsiella pneumoniae* are typically hypermucoviscous, highly tissue-invasive, and capable of causing severe community-acquired infections in otherwise healthy individuals. In contrast, classical (cKp) strains are more often associated with healthcare-acquired infections and exhibit higher rates of antimicrobial resistance. Despite these differences, both types share major virulence determinants, with the capsule polysaccharide (CPS) being a key factor that protects against host immune defenses and supports bacterial survival (Li et al., 2014; Saki et al., 2022).

### Capsular polysaccharide

The thick capsular layer on *K. pneumoniae* surface protects it from opsonization, phagocytosis, and the action of neutrophils, macrophages, epithelial cells, and dendritic cells (Cortés et al., 2002a; Evrard et al., 2010; Pan et al., 2011; Sahly et al., 2000). An increasing level of CPS material in *K. pneumoniae* serotypes like well-known hypervirulent strains K1 and K2 provide a steady escape from the neutrophil-mediated intracellular killing of the bacterium, resulting in abscess formation in the liver (Wu et al., 2010). The K1 serotype belongs to ST57 and ST23, which are placed together in CG23 (Brisse et al., 2009). The STs with the K2 serotype are distributed mostly in CG375, CG380, and CG86 (Bialek-Davenet et al., 2014a).

The presence of RmpA regulator and aerobactin is a characteristic feature of hvKp, both are encoded by virulence harboring plasmids. In addition, yersiniabactin, which is an iron acquisition system is associated with specific hvKp strains as well. It is encoded by ICEKp1 (integrative conjugative element Kp1). It has been demonstrated that hypermucoviscosity has some association with antibiotic resistance as well. Hypermucoviscosity is more common in strains harboring blaSHV and blaTEM (Dong et al., 2022).

Capsules may play a significant role both outside and within the host; they help to avoid desiccation in the atmosphere, prevent complement-mediated lysis or phagocytosis and antibodies neutralization *via* releasing the capsular content (Clements et al., 2008; Cortés et al., 2002a). In *K. pneumoniae* about 80 types have been reported based on antigenic diversity in capsules (Pan et al., 2008; Shon, Bajwa & Russo, 2013), K1 and K2 types are found to be resistant to phagocytes (Shon, Bajwa & Russo, 2013). These specified types may also have a crucial role in virulence as the K2 capsular type has often been detected in clinical iso-lates of urinary tract infections, pneumonia, and septicemia (De Jesus et al., 2015; Hennequin et al., 2012; Turton et al., 2008).

### Iron acquisition systems

The kfu (iron acquisition system) and PTS (phosphoenolpyruvate sugar phosphotransferase system) serve as security pathways for the iron supply which is critically important in pathology associated with tissue-invasive *K. pneumoniae* (Lawlor, O’Connor & Miller, 2007). The siderophores including yersiniabactin, aerobactin, enterobactin, and salmochelin are iron chelators, these elements provide strength to *K. pneumoniae* against iron deficiency (Bachman et al., 2011). Aerobactin may serve as a virulence enhancer (Lawlor, O’Connor & Miller, 2007) and has been reported to be responsible for more than 90% of the siderophore activities in hypermucovisous *K. pneumoniae*. Yersiniabactin has shown the ability to confer and maintain pneumonia and respiratory infection (Bachman et al., 2011).

### Fimbrial Adhesins

Fimbriae is another significant virulence factor associated with infection and biofilm production, *i.e*., type 1, type 3, Kpc, and KPF-28 adhesins. Type 1 fimbriae serve as an initial factor in urinary tract infections (UTIs). However, it has been reported that fimbriae have no role in the colonization of *K. pneumoniae* in the lungs or intestine (Struve, Bojer & Krogfelt, 2009). Type 3 fimbriae have a crucial role in biofilm but have no part in intestine or pulmonary infections. The types 1 and 3 fimbriae both worked in a compensating way and have a significant role in the colonization of *K. pneumoniae* and its biofilm-associated UTI (Struve, Bojer & Krogfelt, 2009). The fimbrial adhesins are frequently associated with hypermucoviscosity in *K. pneumoniae* and play a contributing role in biofilm production (Wu et al., 2010). The KPF-28 adhesins facilitate *K. pneumoniae* colonization in the mammalian intestine (Di Martino et al., 1996). It has been demonstrated that CF29K protein is prevalent in the CC23 and could be either directly associated with pyogenic liver abscess pathogenesis or related to a different virulence factor on that plasmid.

### Outer membrane proteins (OMPs)

Outer membrane protein A (OmpA) is vital for pathogenesis and also has a major role in the immune evasion mechanism exhibited by *K. pneumoniae in vitro* and *in vivo* (March et al., 2011). The OmpA enables the *K. pneumoniae* for host invasion, serum resistance, and protection from lung collections (Sukumaran, Shimada & Prasadarao, 2003). However, OmpA is a target of neutrophil elastases and serum amyloid protein A, which are the components of the innate immune system of the host, leading to cell lysis and enhancing phagocytosis (Belaaouaj, Kim & Shapiro, 2000; Hari-Dass et al., 2005).

### Lipopolysaccharide and lipid A modifications

Lipopolysaccharide (LPS) is essential for the formation of the outer monolayer of the membrane in Gram-negative bacterial pathogen, lipid A moiety modification helps *K. pneumoniae* in the evasion from the innate immune system of the host. There may be some association between lipid A modification and antibiotic resistance in Klebsiella species (Llobet et al., 2015), however, more studies are needed to corroborate this hypothesis. For instance, colistin causes the disruption of the outer membrane by interacting with lipid A. Primarily LPS modification followed by the addition of 4-amino-4-deoxy-L-arabinose to lipid A are the causes of colistin resistance in *K. pneumoniae*. This change is linked with operon pbgPE regulated by PmrAB/PhoPQ, which is determined through the insertional activation of the PhoQ/PhoP MgrB regulators.

### Other virulence genes

Hospital and other health centers acquired infections due to *K. pneumoniae* led the investigators to figure out the contribution of different virulence factors in the progression of disease (De Jesus et al., 2015). These contributors are the fimbrial and non-fimbrial adhesins, a capsule, siderophores (particularly enterobactin), urease, lipopolysaccharide (LPS), serum resistance as well and biofilm formation (Clements et al., 2008; De Jesus et al., 2015; El Fertas-Aissani et al., 2013; Fuursted et al., 2012; Hennequin et al., 2012). On the other hand, enhancement of the features increasing invasion comprises other siderophores (aerobactin and yersiniabactin), catechol receptor, mucoid factor, and hypermucoviscosity (De Jesus et al., 2015; El Fertas-Aissani et al., 2013; Russo et al., 2011; Struve, Bojer & Krogfelt, 2008). *K. pneumoniae* shows a variety of fimbrial and non-fimbrial adhesins having the ability to recognize various cell receptors which in turn can enable it to attach the target cell surfaces (Struve, Bojer & Krogfelt, 2008). Fimbrial adhesins comprised of mannose-sensitive type 1 fimbria, type 3 fimbriae, and plasmid-encoded fimbriae designated as KPF-28, whereas CF29K is a non-fimbrial adhesins (Podschun & Ullmann, 1998; Schroll et al., 2010; Struve, Bojer & Krogfelt, 2008). Type 1 and type 3 fimbriae have frequently been reported in *K. pneumoniae* species, and cause UTIs and biofilm formation (El Fertas-Aissani et al., 2013; Schroll et al., 2010). Fimbrial adhesins are useful as these enhance the adherence capabilities of the pathogen. On the other hand, it can be disadvantageous in the way that it may trigger the immune system of the host indicating the opportunistic nature of *K. pneumoniae* (De Jesus et al., 2015).

The hypervirulent strain of *K. pneumoniae* contains high quantities of siderophores (Shon, Bajwa & Russo, 2013), which are encoded by genes including entB (enterobactin), iutA (Aerobactin), irp1-irp2-ybtS-fyuA (yersiniabactin) and iroN (ferric catecholates receptor) (Turton et al., 2008). The *K. pneumoniae* genome encodes multiple virulence-associated genes with diverse pathogenic functions. For instance, *uge* encodes UDP-galacturonate 4-epimerase, which is involved in capsule and lipopolysaccharide (LPS) synthesis; *wabG* participates in the biosynthesis of the LPS outer core; and *ureA* is part of the urease operon, enabling nitrogen metabolism and promoting bacterial survival in acidic environments. *magA* is associated with K1 capsule formation and is a key determinant in invasive liver abscess pathogenesis, while *allS* regulates allantoin metabolism, enhancing bacterial growth in host tissues. The *rmpA* gene upregulates capsule production, contributing to hypermucoviscosity, and *mrkD* encodes an adhesin located on type 3 fimbriae that facilitates binding to host surfaces. The *kfu* operon functions as an iron acquisition system essential for growth in iron-limited host environments, and *cf29a* encodes a non-fimbrial adhesin frequently linked to liver abscesses (Brisse et al., 2009; Gao et al., 2014). Additionally, acquired β-lactamase encoding genes increase the pathogenicity of *K. pneumoniae*; however, active infection is primarily dependent on a variety of host-dependent factors (El Fertas-Aissani et al., 2013).

## Naturally occurring resistance determinants

**Figure 3:**
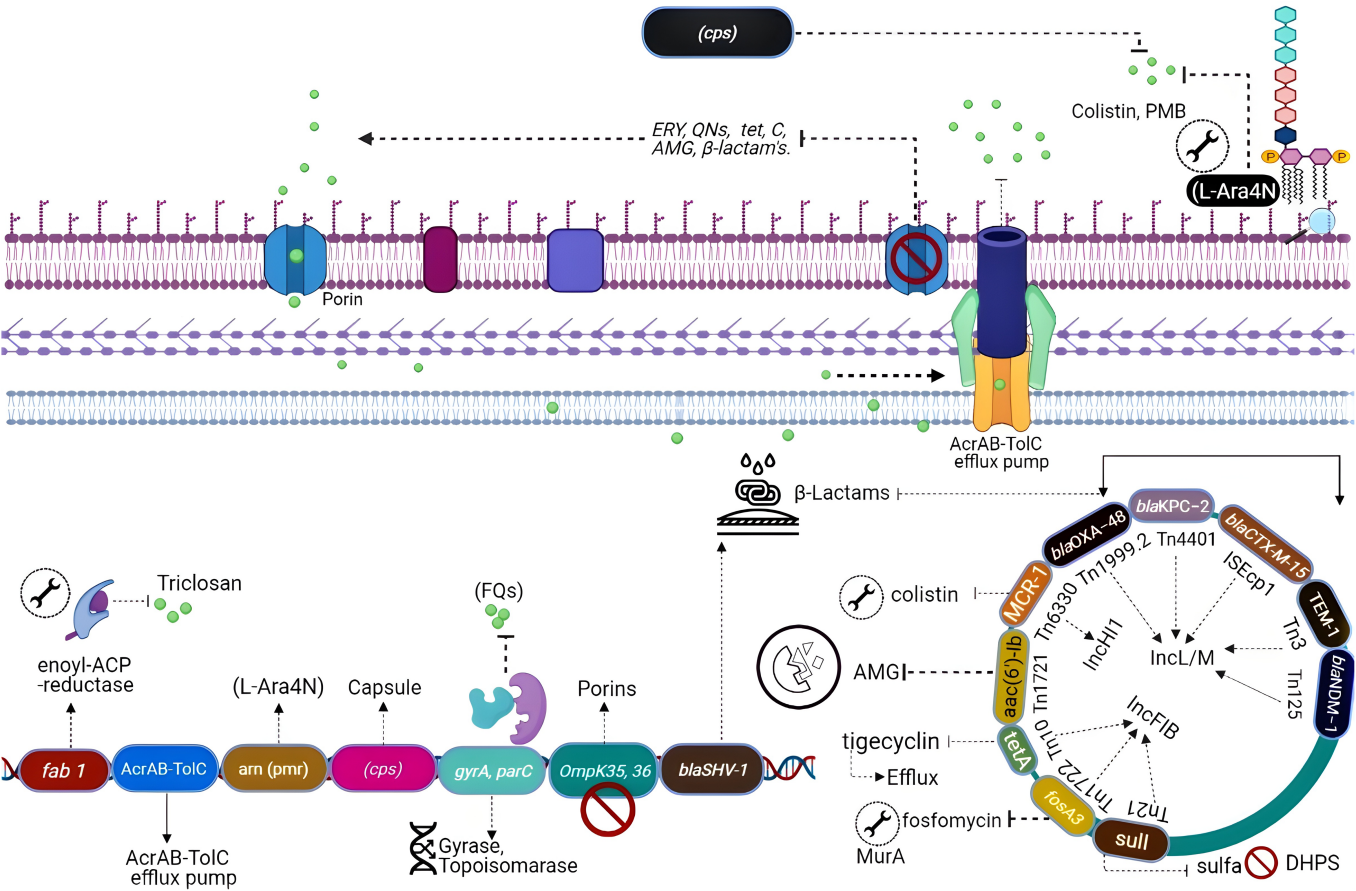
Genetic insights into various resistance mechanisms employed by *K. pneumoniae*.

All the genes that can confer antibiotic resistance when grouped are as resistors (Fig. 3) (Wright, 2007). One of the schemes used for the classification of β-lactamases is molecular classification, based on the amino acid sequences and dividing them into class A, C, and D enzymes that utilize serine, whereas class B metallo-β lactamases require zinc for hydrolysis (Bush & Jacoby, 2010). Formerly, *K. pneumoniae* was the lone Gram-negative enteric bacterium that harbored a chromosome-encoded penicillinase (Arakawa et al., 1986). *K. pneumoniae* exhibits species-specific class A chromosome encoded β-lactamases which cause resistance against ampicillin, carbenicillin amoxicillin, and ticarcillin (Lee et al., 2006). Overall, three different families including SHV, LEN, and OKP have been identified as the source of chromosome-based β-lactamases in *K. pneumoniae*, steer intrinsic resistance to ampicillin *via* the production of class A β-lactamase *e.g*., SHV, encoded by a core gene blaSHV (Holt et al., 2015; Yalew et al., 2022). Two core locus OqxAB (efflux pump) and fosA (glutathione S-transferase) have also been detected in the *K. pneumoniae* chromosome using MGEs and distributed to other bacterial species. The wild-type gene expression of both loci is associated with resistance against fosfomycin (*i.e*., fosA) and quinolones (*i.e*., OqxAB) (Li et al., 2019).

In the mid-20th century, the use of aminoglycosides was replaced by third-generation cephalosporins, carbapenems, and fluoroquinolones (Doi, Wachino & Arakawa, 2016), which resulted in a reduction of novel resistance mechanisms against aminoglycosides. However, the evolution of 16S RNA methylase (Poulikakos & Falagas, 2013) extended the resistance spectrum against all aminoglycosides (Srinivasan & Rajamohan, 2013), whereas kpnEF (SMR-type efflux pump) developed strong resistance against tobramycin and spectinomycin (Naeem et al., 2016). Resistance to tobramycin, streptomycin, and spectinomycin is considered linked directly with the loss of KpnO porins. Mutations in rrs or rpsL, result in target modification augment the resistance patterns (Redgrave et al., 2014). Extensive use of fluoroquinolones after their discovery in the 1980s has directed quinolone resistance mechanisms (Ward-McQuaid, Jichlinski & Macis, 1963). Right after the first use of nalidixic acid (Guerra et al., 1983) and norfloxacin (Guerra et al., 1983), *K. pneumoniae* developed a vast variety of resistance mechanisms against quinolones including target modification *i.e*., gyrA-gyrB subunits and parC-parE subunits of DNA gyrase topoisomerase IV (Martinez-Martinez et al., 1996; Guillard et al., 2016). Other mechanisms include the expression of efflux pumps acrAB gene (Wong, Chan & Chen, 2015) and OmpK36 porins deficiency (Ping et al., 2007).

Polymyxins which perturbs bacterial membrane *via* cations (Ca+2/Mg+2) dislocation are considered as one of the last resort antibiotics against Enterobacteriaceae (Antoniadou et al., 2007). Resistance to colistin was initially reported in 2004 from Greece (Marchaim et al., 2011). Resistance against colistin mainly occurs due to mutation in lpxM and its regulator ramA, responsible for the maturation of lipid A (Marchaim et al., 2011), while the addition of amino arabinose results in neutralization of lipid A. Lipid A modification through TupA-like/glycosyltransferase and CrrAB is also an important resistance mechanism (Srinivasan et al., 2012). Upregulated efflux expression *via* positive regulation of AcrAB-TolC and KpnEF (Lee et al., 2016) by the RarA transcription regulator is imperative. Most commonly the resistance to colistin develops *via* mgrB gene inactivation or point mutations in phoPQ, pmrAB, or crrAB (two-component regulator systems) (Lee et al., 2016).

Additionally, resistance against first approved glycylcyclines *i.e*., tigecycline has also been reported (Nielsen et al., 2014) through modification in the 30S and the 16S ribosomal units and cell permeability (Villa et al., 2014). Other mechanisms include up-regulation of efflux pumps such as KpgABC (Ahn et al., 2016). The first mutation was detected in S10 (ribosomal protein) encoded by rpsJ, which reduces susceptibility, but their role in tigecycline resistance is unclear (Pitout, Nordmann & Poirel, 2015).

## Plasmid-mediated antibiotic resistance

In *K. pneumoniae*, ARGs attained through horizontal gene transfer play a significant role in the acquisition of resistance as compared to chromosomal mutations. Such accessory genes are often plasmid-mediated; however, these may be incorporated into the bacterial chromosome. For instance, a strong promoter enables the mobile genetic variant of blaSHV with some point mutations to perform ESBL activity, which causes resistance against cephalosporins and even carbapenems (Liakopoulos, Mevius & Ceccarelli, 2016). Accordingly, a few *K. pneumoniae* strains cart replicas of blaSHV, one core chromosomal gene, and other acquired plasmid variants directed by a robust IS26 promoter (Hammond et al., 2005).

*K. pneumoniae* can acquire resistance genes reside on plasmids and mobile elements (Bush & Jacoby, 2010; Calbo & Garau, 2015), like blaOXA (Evans & Amyes, 2014), blaPER, blaTLA and blaVEB (Philippon et al., 2016), rare genes blaGES and blaSFO (Ramirez et al., 2019; Yigit et al., 2001). During the 1960s two β-lactamase blaSHV-1 and blaTEM-1 were described in *K. pneumoniae* for the first time which conferred resistance to penicillin (Datta & Kontomichalou, 1965). Later, the acquisition of blaTEM-3 unveiled resistance against mono-bactams and cephalosporins (Sirot et al., 1987).

In the early 2000’s plasmid, plasmid-mediated blaCTX-M shifted the trends of *K. pneumoniae* infections to major hospital-acquired acute infections. It was documentation that metallo-enzyme named blaIMP-1 identified in *K. pneumoniae* displayed resistance to carbapenems. Among other carbapenemases acquired by *K. pneumoniae* including blaNDM-1, blaOXA-48 and blaKPC are the most common and immensely disseminated resistance determinants in every continent (Naas et al., 2012).

Aminoglycosides on the other hand were frequently used during the early 1940s to late 1960 which were then replaced by β-lactams such as cephalosporins and carbapenems as plasmid-mediated resistance determinants like aph, ant, and aac genes were identified against these antibiotics (Navon-Venezia, Kondratyeva & Carattoli, 2017). Unfortunately, Plasmid-mediated aminoglycoside-resistant gene armA is identified, which encodes 16S rRNA methylase enzyme confers resistance to all classes of aminoglycoside, while other 16S rRNA methylase genes belong to the NpmA and Rmt family (Shen et al., 2020).

The very first plasmid-mediated quinolone resistance in *K. pneumoniae* described that qnrA encodes a pentapeptide repeat protein that is responsible for the resistance. Overall, the acquisition of plasmid-mediated resistant genes (PMQR) is associated with resistance to quinolones. These genes include aac (6′)-Ibcr (Bado et al., 2016; Fàbrega et al., 2009; Ruiz et al., 2012) which modifies quinolones in *K. pneumoniae* and qnrA genes whose product protects DNA gyrase and topoisomerase IV from quinolone inhibition in *K. pneumoniae*. PMQR genes modify quinolones in *K. pneumoniae* and pose a narrow spectrum of resistance but their presence augments resistance of *K. pneumoniae* harboring ESBL genes (Tóth et al., 2014). It has been observed in the clonal groups ST11, ST15, and ST147 (Antoniadou et al., 2007).

Plasmid-mediated polymyxin resistance in *K. pneumoniae* strains is also reported in China after the identification of the mcr-1 harboring strains (Zowawi et al., 2015), which modifies lipid A through phosphoethanolamine transferase enzyme activity. Further-more, the recent emergence of hypervirulent colistin resistance *K. pneumoniae* is a major public health concern worldwide keeping in view the colistin as a last resort antibiotic against carbapenem resistance hvKp. However, it is worth mentioning here that mcr-1 is not solely associated with colistin resistance. Other determinants including mcr-2 to 7 and more recently the mcr-8 gene are also associated with colistin resistance in *K. pneumoniae*. Additionally, mcr-7.1 which has 70% amino acid similarity with mcr-3 and mcr-8.1 on a plasmid having IncFIA has been reported as a novel mobile genetic element from various parts of the world (Mmatli, Mbelle & Osei Sekyere, 2022).

The CG 258 harboring *K. pneumoniae* carbapenemase (KPC) was first re-ported from the USA, and blaKPC genes reside in a unique Tn4401 transposon (Naas et al., 2012). Most *K. pneumoniae* plasmids cannot be typed by PCR-assisted replicon typing methods (Osborn et al., 2000). However, many of these novel plasmids are considered to belong to the IncF plasmid family. Based on sequencing data FII replicons of large plasmid family IncFII can be characterized as FIIs, FIIy, and FIIk specific groups (Kaplan et al., 2015). Plasmids also produce an ability to bypass the incompatibility effect where two in-compatible plasmids can reside in the same cell (Chen et al., 2013). This phenomenon is achieved when plasmids replicate using alternative replicons. *K. pneumoniae* strains undergo the recombination of homologous regions of FIIk replicons. ST258 was isolated from the USA in 2000 has blaKPC-2 along with blaKPC-3 encoded by IncFIIk and PKpQIL plasmids.

Phylogenetic studies of CG 258 have demonstrated that plasmids belonging to IncI2 are only present in clade II and pKpQIL were found in both clades I and II (Miriagou et al., 2010). Rearrangements of IncFIIk plasmids portions with IncR or IncN plasmids merged in a multi-replicon status have also been seen. Some other diverse plasmids have been described to have resistance genes like NDM metallo-lactamases (MBL), GES, and the carbapenem-hydrolyzing class D OXA β-lactamases (CHDL) and are disseminated in geologically distant *K. pneumoniae* strains. In Greece, plasmids carrying IncN1 blaVIM-1 were identified from different Klebsiella strains isolated from numerous hospitals containing distinct regions having several transposons and integrons (Poirel et al., 2013). The plasmid IncX3 is highly disseminated in *K. pneumoniae* as it acquires resistance genes including blaNDM-5, (Fig. 3). It has been described that blaCTX-M genes are mostly associated with IncFII plasmids which are related to IncFII of *E. coli* and highly like plasmid IncFII having FIA replicon and the phage P1, adept of extra chromosomal replication by the IncY replicon and diverge from those carrying blaKPC (Dolejska et al., 2013). Plasmids including IncI1, IncR, and IncN are reported as of animal origin while they also acquired CTX-M-15 and CTX-M-1 (Zhu et al., 2009). The data suggests that ESBL-encoding plasmids are highly disseminated within Klebsiella and other Enterobacteriaceae. Interestingly, strains of *K. pneumoniae* isolated from China were carrying pCTX-M-3 plasmid lacking ArmA (Zhu et al., 2009). Overall, taking into consideration IncFIIk plasmids, IncHI, IncI2, and IncN2 alongside novel replicons identified, resistance plasmids of *K. pneumoniae* are distinctive and differ from those which are identified in other members of the Enterobacteriaceae family (Navon-Venezia, Kondratyeva & Carattoli, 2017).

## Infection biology and immune evasion

*K. pneumoniae* prevents the triggering of the host defense mechanism by covering its PAMPs from PRRs, immune globulins, and complement proteins. It prevents binding to both cells of innate and adaptive immunity (Paczosa & Mecsas, 2016). Activation of complement proteins by *K. pneumoniae* occurs in antibodies independent manner as it binds directly to Cq1 (Albertí et al., 1996; Alberti et al., 1993). Although *K. pneumoniae* also activates the complement classical pathway by binding of LPS to complement protein. However, this mechanism of activation was reported as less efficient as compared to outer membrane proteins (Alberti et al., 1993). The complement system plays a crucial role in phagocytosis and clearance of *K. pneumoniae* by lung epithelial cells facilitated by the C3b complement protein (de Astorza et al., 2004). Mutation of capsular polysaccharides ultimately increases the C3b deposition, which results in string bactericidal activity complement proteins. To avoid increased deposition of C3b, the O antigen and LPS of outer membrane work as a shielding factor (Merino et al., 1992). Other than LPs and O antigen, CPS also inhibits complement deposition (Álvarez et al., 2000) and inhibits binding of lung collectins SPA and SP-D to LPS. Studies conducted on mouse models strongly fortify the argument that CPS plays a crucial role in *K. pneumoniae* virulence (Willsey et al., 2018) by inhibiting the binding of polymyxins and CAMP therefore, it has been stated that resistance to polymyxins is directly proportional to the amount of CPS produced by *K. pneumoniae* (Campos et al., 2004). Another mechanism to invade CAMPs and Polymyxins includes modification in Lipid A structure (Llobet, Tomas & Bengoechea, 2008). The absence of palpitate, 4-amino-4-deoxy-L-arabinose, phospho-ethanolamine, and 2-hydroxy myristate from Lipid A structure results in loss of virulence in mouse models (Kidd et al., 2017; Llobet et al., 2011; Mills et al., 2017). However, something worth mentioning here is that the role of CPS in virulence is indirect as level CPS depends upon 2-hydroxylation and switches on the status of late acyltransferases lpxM and lpxL respectively (Llobet et al., 2011).

It has been reported that *K. pneumoniae* invades the effect of antibiotics and the immune system by penetrating epithelial cells (Clements et al., 2007). However, further research on this phenomenon revealed that the engulfment of *K. pneumoniae* by host epithelial cells is a defense mechanism (Clements et al., 2007). *K. pneumoniae* CPS agonistically activates the TLRs especially the TLR4 function which results in an enhanced inflammatory effect as the number of TLR4 and TLR2 increase in epithelial cells because of *K. pneumoniae* infection (Cortés et al., 2002b). The host immune system also produces anti-CPS immunoglobulins which activate the secretion of neutrophil extracellular traps (NETs), which upon release kills *K. pneumoniae* in extracellular space (Regueiro et al., 2009). Phosphatidylserine is known as the ‘eat me’ signal for macrophages; however, their reduced expression of neutrophils because of their infection ultimately inhibits their phagocytosis (Diago-Navarro et al., 2018) and leads them towards necroptosis and inhibits efferocytosis of neutrophils (Amulic et al., 2012). Subsets of dendritic cells are also activated by *K. pneumoniae* (Jondle et al., 2018), while structures including CPS, LPS, and porins, induce their maturation (Jondle et al., 2018). Inside macrophages, *K. pneumoniae* controls the phagosome maturation and 10 h after *K. pneumoniae* infection programmed cell death of macrophages usually occurs (Van Elssen et al., 2010). Interestingly, there is no evidence that CPS augments the *K. pneumoniae* survival inside macrophages, as CPS mutants do not affect intracellular survival patterns, supported by the fact that *K. pneumoniae* inhibits its CPS production once it gets inside the cell (Van Elssen et al., 2010). The plasticity of macrophages allows them to have physiological and phenotypical characteristics. Studies have demonstrated the M2 macrophage presence in mouse infection models, while the elimination of M2 macrophages results in efficient clearance of pathogen (Mills et al., 2017).

High levels of IL-10 during *K. pneumoniae*-triggered pneumoniae result in an anti-inflammatory effect (Fevre et al., 2013). IL-10 cytokines are used to control the activation of cells involved in innate immune response and are secreted by various immune cells (Yoshida et al., 2000). To counter this, the *K. pneumoniae*-induced anti-inflammatory affect mediated by IL-10 host immune system regulates IFNγ production (Gabryšová et al., 2014). Reports also claim the direct association between CPS and high levels of IL-10 fortifies the pathogenicity of *K. pneumoniae*, while mice infected with mutant CPS do not have high IL-10 concentrations (Gabryšová et al., 2014). NF-κB (transcription factor), upon stimulation of a TLR4/2-MyD88 signaling pathway, controls various anti-Klebsiella responses (Yoshida et al., 2001). Here CPS comes into play by inhibiting the engulfment of *K. pneumoniae* by epithelial cells resulting in limited NF-κB activation which in turn further suppresses the production of IL8, ICAM1, and human defensins. In deubiquitinase cylindromatosis (CYLD) negative host cells Klebsiella infection quickly followed by production of IL8 this happens because in (CYLD) positive cells *K. pneumoniae* hijacked the (CYLD) thus inhibits NF-κB signaling (Bengoechea & Sa Pessoa, 2019). Studies have shown CPS mutants are unable to activate the EGFR pathway, while CPS wild strain does (Bengoechea & Sa Pessoa, 2019). However, their activation is indirect and TLR4-dependent (Moranta Mesquida et al., 2018). *K. pneumoniae* inhibits the production of inflammatory mediators and defensins by inactivating the MAPK-by-MAPK phosphatase-1 (MKP-1). As MAPKs p38, ERK and JNK play important roles in the inflammatory response. The production of (MKP-1) during infection is mediated by activation of NOD1, while inhibition of IL8 from epithelial cells is governed by the synergistic effect of MKP-1 and CYLD (Regueiro et al., 2011). Studies have confirmed the CPS-independent anti-inflammatory role of OmpA during *K. pneumoniae* infections (Tomás et al., 2015).

**Figure 4:**
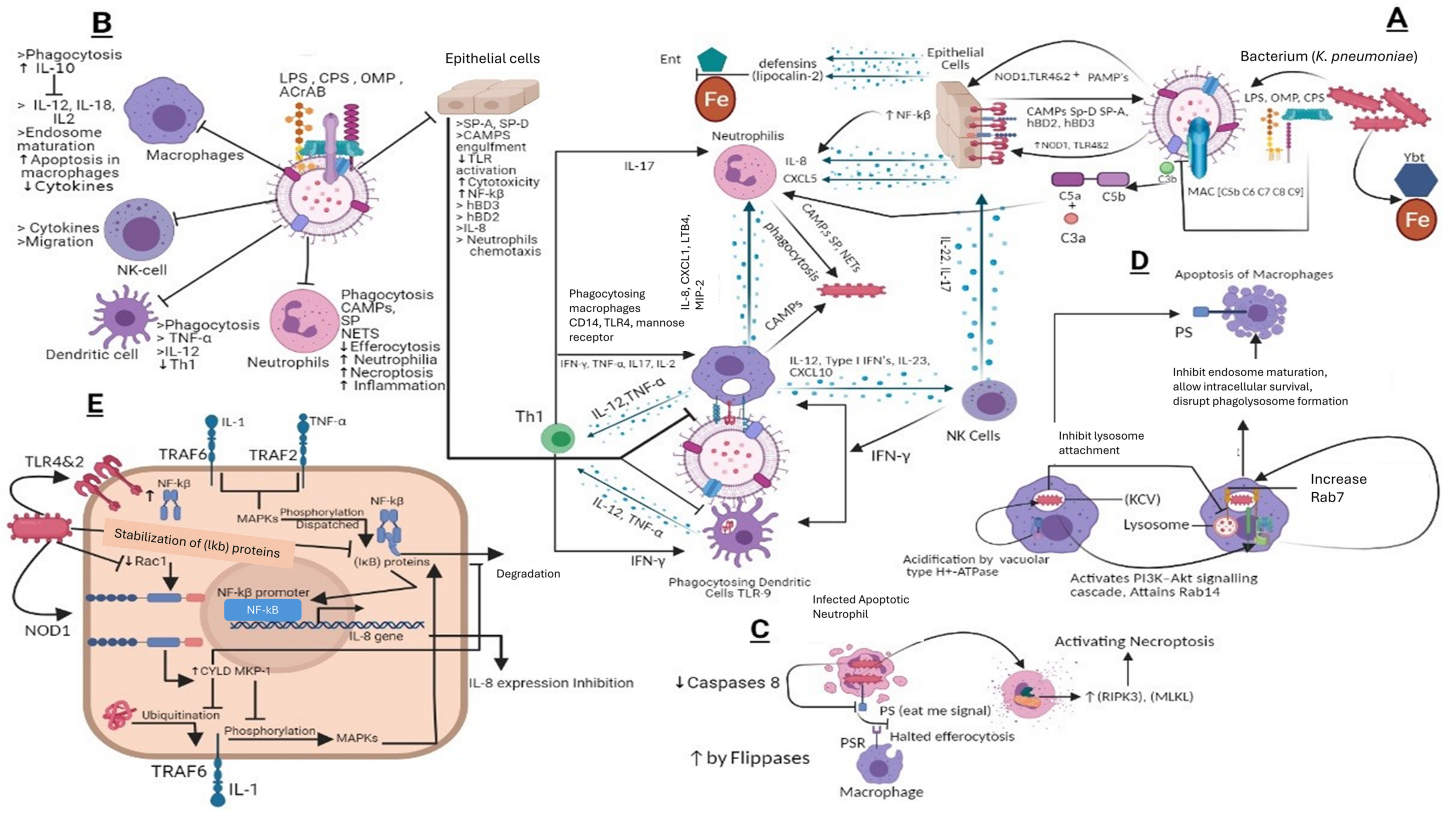
(A) Host immune strategies against *K. pneumoniae*. *K. pneumoniae* elicits host innate immune response right after its encounter with respiratory epithelial cells and soluble effectors of the immune system facilitated through several immunologically and pathogenically crucial structures that include LPS, CPS, OMP, AcrAB, PPG, (PAMPs) whose interaction with PRRs like TLR2, 4 and 9 laid the foundations of *K. pneumoniae* pathogenesis. In response to *K. pneumoniae* active infection complement associated with C3b deposition, NF-kβ mediated activity of IL-8 for leukocyte recruitment, macrophages and dendritic cells TLR4, TLR9 dependent phagocytic and inflammatory activity and NK cells recruitment through IL-23 and siderophores inhibitors collectively restrains the lethal outcomes of *K. pneumoniae* infection. (B) *K. pneumoniae* host evasion strategies. *K. pneumoniae* virulence trove plays crucial role in abrogating host immune mechanism. This arsenal includes, LPS, CPS, OMP, and AcrAB inhibits phagocytosis from dendritic cells, macrophages, neutrophils and epithelial cells. CPS also inhibits SP-A and SP-D and C3b deposition, MAC, and CAMP. This ultimately inhibits Th1 activation, which is considered as bridge between innate and adaptive immunity. CPS and LPS mediated evasion of phagocytosis halts the activation of NK cells by IL23 and Type 1 IFNs. (C) *K. pneumoniae* mediated inhibition of efferocytosis and activation of necroptosis in neutrophils. *Klebsiella pneumoniae* down regulates caspases which when activated restricts flipases to retain PS (‘eat me’ signal for macrophages) signal inside the infected cell. This activity of flippase to inhibit PS exposure to exoplasm inhibits efferocytosis of apoptotic neutrophils. Further *K. pneumoniae* activates necroptosis by a programmed cell death which is mediated by receptor interacting protein kinase-3 (RIPK3) and its substrate mixed lineage kinase like (MLKL). (D) *K. pneumoniae* survival within macrophages and apoptosis in macrophages. *K. pneumoniae* after its engulfment mediated by PI3K, survives within (KCV). *K. pneumoniae* manages to survive within endosome by preventing its fusion with lysosome by activating PI3K–Akt signaling cascade and localizing LAMP1 and Rab7 But negative for fusion markers like cathepsin D, TR‐dextran. Acidification through H+‐ATPase. While apoptosis of is initiated through PS. (E) Klebsiella antagonizes the activation of NF‐κB *via* the deubiquitinase CYLD and blocks the phosphorylation of MAPKs. *K. pneumoniae* inhibits pro inflammatory transcriptional factor NF-kβ for IL-8 expression by activating CYLD and MKP‐1 *via* Rac1 inhibition leads to NOD1 activation manner. CYLD inhibits the ubiquitination of TRAF6 while MKP-1 inhibits phosphorylation of MAPK which otherwise phosphorylates and dispatch the NF‐κB inhibitory proteins leads to NF-kβ activation. LPS, Lipopolysaccharides; OMP, Outer membrane proteins; CPS, Capsular polysaccharides; ACrAb, Efflux pumps; MAC, membrane attack complex; PAMPs, pathogen‐associated molecular patterns; NOD1, Nucleotide-binding oligomerization domain-containing protein 1; NLR, nucleotide binding and oligomerization domain‐like receptors; TLR, Toll like receptors; SP, Surfactant proteins CAMPs; Cationic antimicrobial peptides, hBD, Human beta defensins; C3b, Complement component 3; C3a&C5a, Anaphylatoxins; NF-kβ, Nuclear factor kappa-light-chain-enhancer of activated B cells; Ent, Enterobactin, Ybt, Yersiniabactin; Fe, Iron; IL-8, Interleukin 8; IL-17, Interleukin 17; IL-22, Interleukin 22; IL-12, Interleukin 12; IL-2, Interleukin 2; IL-10, Interleukin 10; IL-1, Interleukin 1; CD14, cluster of differentiation 14; CXCL5,1&10, Chemokines; TNF-α, Tumor necrosis factor alpha; IFN-γ, Interferon gamma; NETs, Neutrophil extracellular traps; KCV, Klebsiella containing vacuole; PS, phospholipid phosphatidylserine; PSR, phospholipid phosphatidylserine receptor; RIPK3, receptor interacting protein kinase-3; MLKL, mixed lineage kinase like; MAPKs, mitogen‐activated protein kinases; CYLD, conserved cylindromatosis codes for a deubiquitinating enzyme; PI3K, phosphoinositide 3-kinase; EEA1, early endosome antigen 1; Lamp1, Lysosome‐associated membrane protein 1; Akt, Protein kinase B; Rab, GTPase; (IκB) proteins, NF‐κB inhibitory proteins; TRAF, (TNF) receptor-associated factors; Rac1, Rho family of GTPases. NK cells, natural killer cells; PPG, peptidoglycans; LTB4, Leukotriene B4; Th1, Type 1 T helper cells; MIP-2, Macrophage Inflammatory Protein-2. This figure was developed using Bio-render (trial version).

Enterobactin is an iron-binding siderophore secreted by *K. pneumoniae* it competes and binds the iron against host proteins (March et al., 2011). Other iron-binding proteins include aerobactin, salmochelin, and yersiniabactin (Bachman et al., 2012). Importantly, yersiniabactin is associated with invasive infections. During *K. pneumoniae* infection the spread of the pathogen is associated with siderophores as they down-regulate transcription factor HIF-1α responsible for mucosal immunity and cellular intrinsic immunity (Holt et al., 2015) the hypothesis that HIF-1α down-regulation increases the infection rate is usually common in Klebsiella infections (Holden et al., 2016). Overall, the immune evasion strategies of *K. pneumoniae* mechanisms are portrayed in Fig. 4.

## Prospectives

*K. pneumoniae*-associated hospital-acquired infections cannot be easily differentiable from HAIs caused by other clinically important pathogens, whereas community-acquired infections caused by *K. pneumoniae* show some distinguished characteristics. Conventionally, infection caused by *K. pneumoniae* is designated as community-acquired pneumonia and clinically manifested as sudden onset of high fever, dramatic toxicity, hemoptysis and abnormalities seen in chest radiography such as bulging interlobar cleft and cavitary abscesses (Ashurst & Dawson, 2023; Korvick et al., 1991). A considerable proportion of some ESBL-producing clinical isolates of *K. pneumoniae* are sensitive to third generation cephalosporins or aztreonam and therefore ESBLs in clinical isolates is problematic (Paterson & Bonomo, 2005; Wang et al., 2011). This confusion results in serious health hazards when the same treatment is used against serious infections (Paterson et al., 2001; Paterson & Yu, 1999), whereas resistance to Ceftazidime is a sufficient marker for the detection of ESBLs.

The Clinical and Laboratory Standards Institute (CLSI) (2008) has standardized confirmatory and screening tests for *K. pneumoniae* and *K. oxytoca* for ESBL detection. Production of some important enzymes including extended-spectrum ß-lactamases, cephalosporinases, and carbapenemases and their continuous horizontal gene transfer *via* plasmids and mobile elements like transposons facilitates the ESBL’s associated infection and bacterial survival under the action of ß-lactam drugs (Partridge et al., 2018). As resistance against known antibiotics keeps on increasing and there is a scarcity of new antibiotics, alternative therapeutic and diagnostic strategies may be exploited (Lewis, 2017). Various detection methods for ESBL have been employed in laboratories that include beta-lactamase inhibitors such as clavulanic acid by using double disk diffusion test, microscan ESBL plus detection system, Vitek ESBL detection card, E test strips containing ceftazidime or cefotaxime (Singh & Singh, 2014). Additionally, a bacteriophage-based diagnostic approach is also practiced. Recently, studies demonstrated a luminescent bacterio-phage-based detection of *K. pneumoniae*, and they suggested that such a diagnostic approach may provide a prompt diagnostic tool to escort the developing subject of phage therapeutics, especially to treat chronic infectious diseases.

While considering novel treatments against drug resistance *K. pneumoniae*, phage therapy is considered a promising therapeutic strategy to fight resistant superbugs. The endolysins that are phage hydrolases and other phage proteins are potential antimicrobials (Aslam et al., 2021a; Qurat-ul-Ain et al., 2021; Zelcbuch et al., 2021). Despite the advancements in this field few challenges still need to be addressed for the general application of phage therapeutics. These shortfalls include target specificity, penetration abilities, immunogenicity, and half-life of the phage product (Karimi et al., 2016).

Immunotherapy represents a promising alternative for managing MDR *K. pneumoniae* by leveraging the host’s immune response rather than relying on antibiotics. This method employs various mechanisms to protect the host and avoid the development of resistance, unlike antibiotics. Practically, an all-in-one vaccine having a complete range of CPS or LPS is difficult, though a multivalent vaccine has been developed. It is suggested that a solution to this problem is to identify conserved antigenic regions among various serotypes of *K. pneumoniae* which may be used for the development of a broad-spectrum vaccine (Xiao, Wu & Dall’Acqua, 2016). In this regard, MrkA is a suitable candidate as it is conserved among various members of the Enterobacteriaceae family is a key element fimbrial (Type III) complex, and possesses key vital functions like biofilm formation, infection progression, and fimbrial shaft development (Allen, Gerlach & Clegg, 1991). Poly-N-acetyl glucosamine (PNAG) is another possible conserved surface polysaccharide antigen that may also be beneficial to manage *K. pneumoniae via* immunotherapy (Cywes-Bentley et al., 2013; Xiao, Wu & Dall’Acqua, 2016). Previously, the vaccine was developed from hyper-immune globulins and capsular polysaccharides of *K. pneumoniae*, but the complexity of its production halted further progress (Ahmad et al., 2012a; Diago-Navarro et al., 2017). In 2017, Diago-Navarro et al. (2017) isolated Monoclonal antibodies against hyper-mucoid hypervirulent strains which promoted the neutrophil extracellular trap (NET) release and opsonophagocytic killing. In preclinical models’ immunogenicity of macromolecules like LPS O antigens tends to increase when conjugated covalently with variety of carriers like outer membrane proteins (Ahmad et al., 2012b). Recently, a humanized anti-body against galactan III O antigen, expressed in about 83% of the surface polysaccharides, has been reported these sugars are optimal targets for the development of immune prophylactic and therapeutic efforts to counter the emergence of antibiotic-resistant strains, along with the hypervirulent ST258 (Szijártó et al., 2017). Diago-Navarro et al. (2018) have also generated murine-based monoclonal antibodies against ST 258 CPS.

In addition to immunotherapy-based approaches, gene-editing tools such as CRISPR-Cas have emerged as promising alternatives for combating multidrug-resistant *K. pneumoniae*. This technology allows the development of sequence-specific antimicrobials, in which a guide RNA directs nuclease activity to precisely target resistance genes, virulence determinants, or other specific DNA sequences, enabling selective bacterial eradication without disrupting the broader microbiota (Pursey et al., 2018; Owaid & Al-Ouqaili, 2025). Guide RNA is delivered proficiently to the target microbial community through phagemid or bacteriophage. The specific DNA targets include polymorphism, virulence determinants, and antibiotic-resistance genes. The application of this approach against *E. coli* and carbapenem-resistant Enterobacteriaceae has been reported in recent studies (Tagliaferri et al., 2020). RNA-guided nucleases (RGNs) are highly specific CRISPR–Cas-based molecular tools in which a guide RNA directs the Cas nuclease to introduce double-stranded breaks at target DNA sequences, enabling sequence-specific bacterial killing or plasmid elimination. Unlike conventional antibiotics, RGNs can be programmed to recognize genetic signatures such as antibiotic resistance genes, virulence determinants, or even single-nucleotide polymorphisms. For example, RGNs delivered *via* bacteriophage can selectively eliminate *E. coli* harboring the *bla*_*NDM*-1_ carbapenem-resistance gene, while sparing non-target bacterial strains, thus allowing precise modulation of microbial communities (Citorik, Mimee & Lu, 2014).

## Conclusion

*Klebsiella pneumoniae* is a notable member of the ESKAPE group of Gram-negative pathogens, distinguished by its diverse repertoire of antimicrobial resistance genes, virulence determinants, complex genomic architecture, and substantial plasmid burden. These features contribute to its remarkable adaptability and therapeutic recalcitrance. Despite extensive research, significant gaps remain in our understanding of its pathobiology, population-level transcriptomics, and mechanisms driving the emergence and dissemination of multidrug resistance. Future studies should integrate advanced genomic, transcriptomic, and proteomic approaches to identify novel therapeutic targets, explore host–pathogen interactions in greater depth, and develop innovative treatment strategies, including precision antimicrobials and immune-based interventions. Such efforts will be crucial to outpace the rapid evolution of this formidable pathogen and mitigate its growing clinical and public health impact.

## Additional Information and Declarations

### Data Availability

The following information was supplied regarding data availability:

This is a literature review.
